# Supplementary material for: When Should Lymphadenectomy Be Performed in Non-Metastatic Pancreatic Neuroendocrine Tumors? A Population-Based Analysis of the German Clinical Cancer Registry Group
Source: Cancers (Basel). 2024 Jan 19;16(2):440. doi: 10.3390/cancers16020440 (PMC10814740; doi:10.3390/cancers16020440)
Supplement: Supplementary file 1 [file cancers-16-00440-s001.zip › cancers-2785362-supplementary.pdf]

Supplementary Materials

When Should Lymphadenectomy be Performed in Non-Metastatic Pancreatic Neuroendocrine Tumors? A Population-Based Analysis of the German Clinical Cancer Registry Group

Thaer S. A. Abdalla [1,*] , Louisa Romina Bolm [1], Monika Klinkhammer-Schalke [2], Sylke Ruth Zeißig [2,3], Kees Kleihues van Tol [2], Peter Bronsert [4], Stanislav Litkevych [1], Kim Christin Honselmann [1], Rüdiger Braun [1],Judith Gebauer [5], Richard Hummel [6], Tobias Keck [1,*], Ulrich Friedrich Wellner [1] and Steffen Deichmann [1]

[1] Department of Surgery, University Medical Center Schleswig-Holstein, Campus Lübeck, Lübeck, Germany
[2] Network for Care Quality and Research in Oncology (ADT), German Cancer Registry Group of the Society of German Tumor Centers, Berlin, Germany
[3] Institute of Clinical Epidemiology and Biometry (ICE-B), University of Würzburg, Würzburg, Germany
[4] Department of Pathology University Medical Center Freiburg, Freiburg, Germany
[5] Department of Internal Medicine I, University Medical Center Schleswig-Holstein, Campus Lübeck, Lübeck, Germany
[6] Department of Surgery, University Medical Center Greiswald, Greifswald, Germany
* Correspondence: thaer.abdalla@uksh.de (T.S.A.A.); tobias.keck@uksh.de (T.K.)

Citation: To be added by editorial staff during production.

Academic Editor: Firstname Lastname

Received: 6 December 2023
Revised: 6 January 2024
Accepted: 17 January 2024
Published: 22 January 2024

Copyright: © 2024 by the authors. Licensee MDPI, Basel, Switzerland. This article is an open access article distributed under the terms and conditions of the Creative Commons Attribution (CC BY) license (https://creativecommons.org/licenses/by/4.0/).

Table S1: Risk of LNM according to tumor size, tumor grade and location.

.Size	Grade	Pancreatic Head	Pancreatic Body/ Tail	N
T1	G1	4.4%	1.3%	90 vs. 158
T1	G2	18.2%	6.4%	11 vs. 47
T1	G3	100%	50%	1. vs. 2
T2	G1	19.5%	6.9%	41 vs. 72
T2	G2	34.2%	26.2%	38 vs. 61
T2	G3	100%	75%	5 vs. 4
T3-4	G1	64%	18%	39 vs. 39
T3-4	G2	54.8%	51.4%	62 vs.70
T3-4	G3	57.7%	57.1%	26 vs 14

Legend: Colors defining different risk of LNM. Green; low-risk (<5%). Yellow; moderate-risk (5-10%). Blue; high-risk (>10%).

Table S2: Frequency of local recurrence and metastasis according to LNM and tumor size

	pNET <2cm			pNET ≥2cm		
**Variable**	***N0***	***LNM***	***p***	***N0***	***LNM***	***p***
**Local recurrence**			0.646			0.034
No recurrence	380 (99%)	20 (100%)		357 (95%)	208 (92%)	
recurrence	4 (2%)	0 (0%)		19 (5%)	18 (8%)	
**Distant metastasis (Progression)**			<0.031			<0.001
No distant metastasis	318 (97%)	14 (87%)		246 (81%)	118 (62%)	
Distant metastasis	9 (3%)	2 (13%)		58 (19%)	67 (37%)	

Legend: *p* according to the χ2 test when comparing patients with and without lymph node metastasis (LNM).

Figure S1: Flow Chart

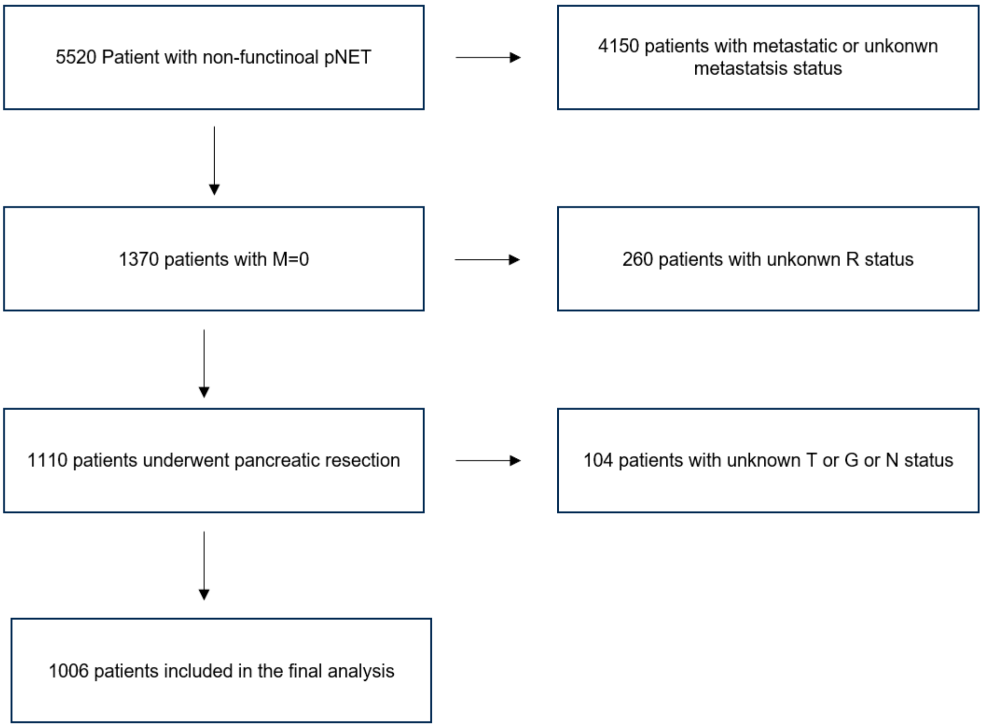

Figure S2: Kaplan-Meier Plot for disease-free survival according to the presence of lymph node metastasis stratified by the T-stage in patients with pNET.

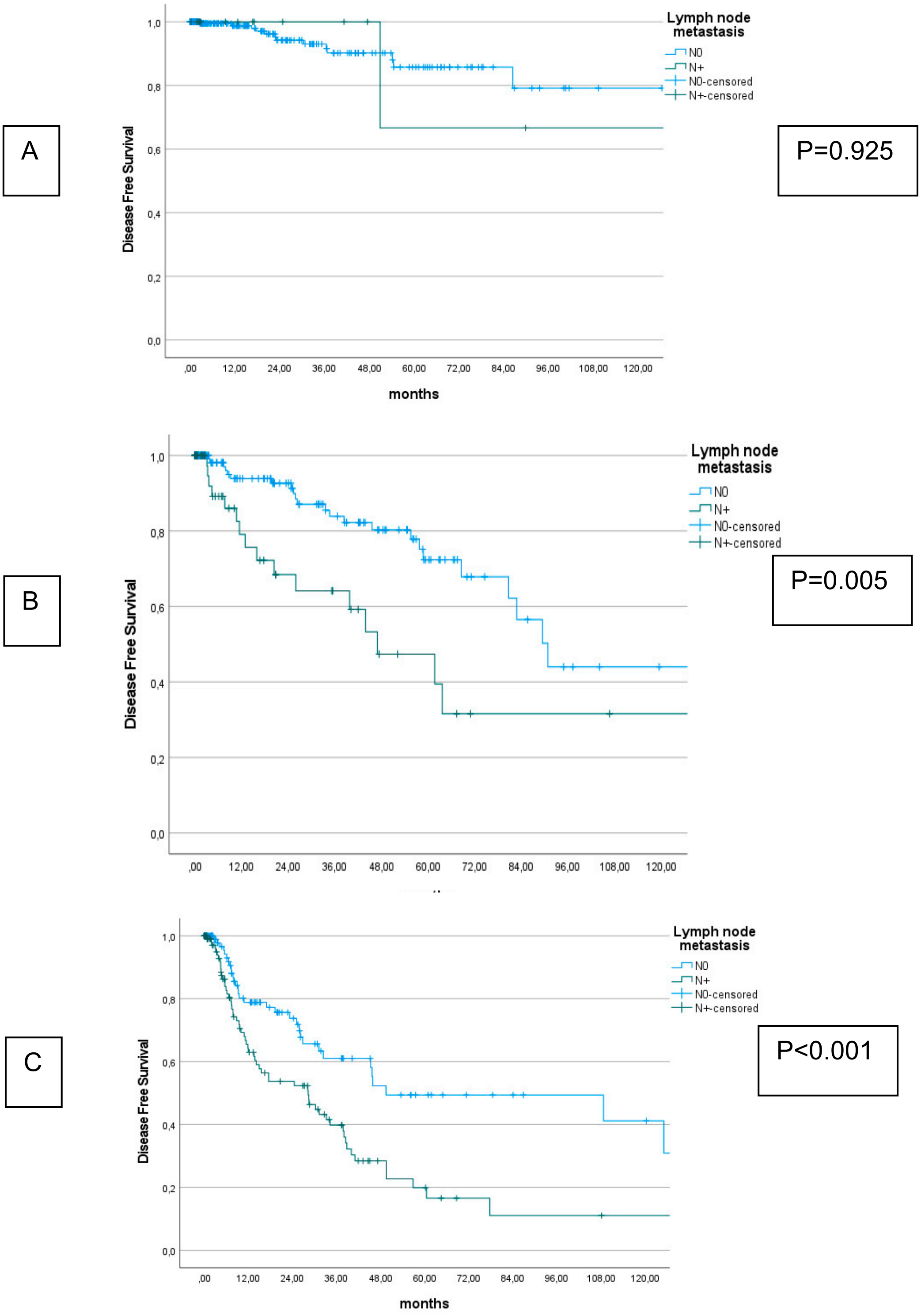

Legend: P according to Log-Rank Test, Figure A; T1 Tumors, Figure B; T2 Tumors, Figure C; T3-T4 Tumors.
